# Implantable Polymeric Drug Delivery Devices: Classification, Manufacture, Materials, and Clinical Applications

**DOI:** 10.3390/polym10121379

**Published:** 2018-12-12

**Authors:** Sarah A. Stewart, Juan Domínguez-Robles, Ryan F. Donnelly, Eneko Larrañeta

**Affiliations:** School of Pharmacy, Queen’s University Belfast, 97 Lisburn Road, Belfast BT9 7BL, UK; sstewart35@qub.ac.uk (S.A.S.); j.dominguezrobles@qub.ac.uk (J.D.-R.); r.donnelly@qub.ac.uk (R.F.D.)

**Keywords:** implants, drug delivery, design, manufacture

## Abstract

The oral route is a popular and convenient means of drug delivery. However, despite its advantages, it also has challenges. Many drugs are not suitable for oral delivery due to: first pass metabolism; less than ideal properties; and side-effects of treatment. Additionally, oral delivery relies heavily on patient compliance. Implantable drug delivery devices are an alternative system that can achieve effective delivery with lower drug concentrations, and as a result, minimise side-effects whilst increasing patient compliance. This article gives an overview of classification of these drug delivery devices; the mechanism of drug release; the materials used for manufacture; the various methods of manufacture; and examples of clinical applications of implantable drug delivery devices.

## 1. Introduction

The oral route remains the most popular and convenient method of drug delivery, with many advantages. However, along with other common routes such as transdermal, or intravenous (IV) injection, it also presents a number of disadvantages and challenges. Many drugs are not suitable for delivery *via* the oral route. This may be as a result of: drug degradation in the acidic conditions of the stomach or alkaline conditions in the intestine [1]; first pass metabolism; or compliance issues. In addition, many newly discovered drug compounds do not possess the ideal chemical properties for oral delivery.

IV delivery may overcome some of the issues associated with oral delivery such as: first pass metabolism; degradation in the stomach; or poor solubility and bioavailability. However, this route is not without disadvantages. A healthcare professional will be required for administration, and there may be sterility or specific storage issues. It is also an invasive delivery technique which may be unsuitable for those patients who suffer from needle phobia. The transdermal route offers advantages such as: avoidance of first pass metabolism; avoidance of gastro-intestinal degradation; and non-invasiveness [2]. However, it also has a number of issues which prevent it becoming the ideal drug delivery route. Very few drug compounds possess the specific properties such as: low molecular weight; a Log P value between 1 and 3; good solubility; and high partition coefficient [2], that are required to pass through the skins outer barrier, the *stratum corneum*, without help from additional methods such as the use of iontophoresis or microneedles.

Therefore, there is a need for novel drug delivery systems to improve delivery of existing drug compounds, and to allow delivery of newly discovered drugs with less than ideal properties for oral drug delivery [3]. The development of new drug delivery systems should aim to optimise effectiveness and tolerability of drug compounds, whilst ideally simplifying their administration [4].

A promising alternative delivery method is the use of polymeric implantable devices to deliver drug compounds. Implantable drug delivery systems allow targeted and localised drug delivery and may achieve a therapeutic effect with lower concentrations of drug [3,5,6]. As a result, they may minimise potential side-effects of therapy, while offering the opportunity for increased patient compliance [7]. This type of system also has the potential to deliver drugs which would normally be unsuitable orally [6], because it avoids first pass metabolism and chemical degradation in the stomach and intestine, thus, increasing bioavailability [7]. Implantable devices will require a healthcare professional for insertion, and the insertion itself will be a relatively invasive process. However, unlike other methods this will only be required once. The prolonged drug delivery that will be achieved without the reliance on patient compliance overcomes these disadvantages. Another advantage of implantable drug delivery devices is that they offer the opportunity for early removal if adverse effects require termination of treatment [8,9].

## 2. Implantable Polymeric Drug Delivery Device Classification

Implantable drug delivery device classification is not a straightforward task as there are a number of complex implants that will fall into hybrid categories. Nevertheless, implantable drug delivery devices can be broadly classified in two main groups: passive implants and active implants. The first group includes two main types of implants: biodegradable and non-biodegradable implants. On the other hand, active systems rely on energy dependent methods that provide the driving force to control drug release. The second group includes devices such as osmotic pressure gradients and electromechanical drives. However, the latter are normally metallic implants and this review focuses on polymeric devices. Consequently, they will not be covered in this review.

### 2.1. Passive Polymeric Implants

These are normally relatively simple devices with no moving parts, they rely on passive diffusion for drug release. They are generally made of drugs packed within a biocompatible polymer molecule. Several parameters such as: drug type/concentration, polymer type, implant design and surface properties can be modified to control the release profile. Passive implants can be classified in two main categories: non-biodegradable and biodegradable systems.

#### 2.1.1. Non-Biodegradable Polymeric Implantable Systems

**Figure 1:**
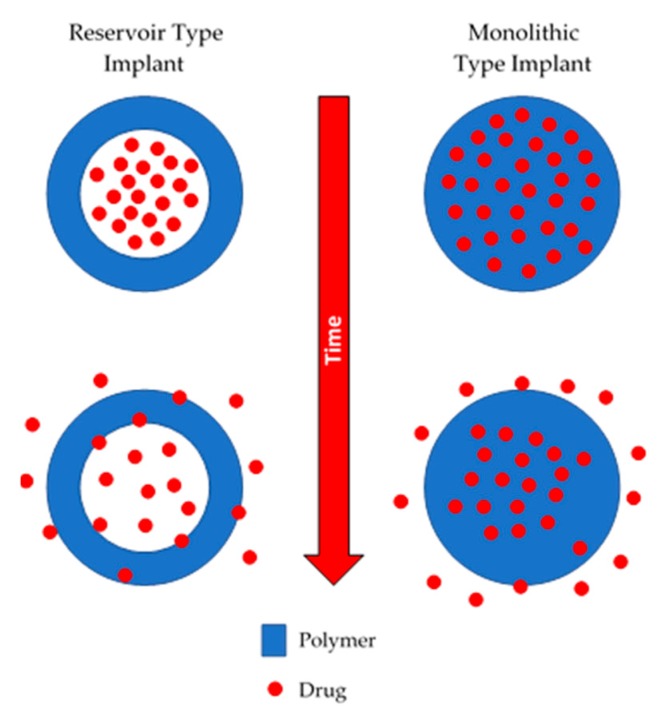
An illustration of reservoir and monolithic type implants.

Non-biodegradable implants are commonly prepared using polymers such as silicones, poly(urethanes), poly(acrylates) or copolymers such as poly(ethyelene vinyl acetate) [10,11,12,13,14]. This type of device can be monolithic or reservoir type implant, as shown in Figure 1. Monolithic type implants are made from a polymer matrix in which the drug is homogeneously dispersed [15]. On the other hand, reservoir-type implants contain a compact drug core covered by a permeable non-biodegradable membrane. The membrane thickness and the permeability of the drug through the membrane will govern the release kinetics [16].

Non-biodegradable implantable drug delivery systems have been extensively used for contraceptive delivery [15]. These devices are structurally resilient and robust over their lifetime. Accordingly, the main drawback of non-biodegradable implants is that after depleting their drug load, they need to be removed. The materials used to prepare these devices show good long-term biocompatibility, but sometimes they can cause infections, tissue damage or cosmetic disfigurement [15]. Accordingly, once all the drug has been released, they are normally extracted to prevent any adverse effects.

#### 2.1.2. Biodegradable Polymeric Implants

Biodegradable implants were developed to overcome the drawbacks of non-biodegradable implants. These devices are made using polymers or block copolymers that can be broken down into smaller fragments that will be subsequently excreted or absorbed by the body [17,18]. Normally they are made using polymers such as poly(caprolactone) (PCL), poly(lactic acid) (PLA) or poly(lactic-co-glycolic acid) (PLGA) [15]. These materials have been extensively studied and their degradation kinetics can be easily tuned to adjust the drug release rate. The main advantage of this type of implant is that they do not need to be extracted after implantation, as they will be degraded by the body of the patient. They can be manufactured using the same designs described in the previous section: monolithic implants and reservoir-type implants [15]. One drawback of this particular type of device is that they are more complex to develop than the non-biodegradable ones. The range of potential materials that can be used is reduced, and the regulatory requirements are stricter as the material will be left behind in the body. Finally, the degradation of the polymeric matrix is the main driving force for drug release. However, this can be highly variable in each patient.

### 2.2. Dynamic or Active Polymeric Implants

**Figure 2:**
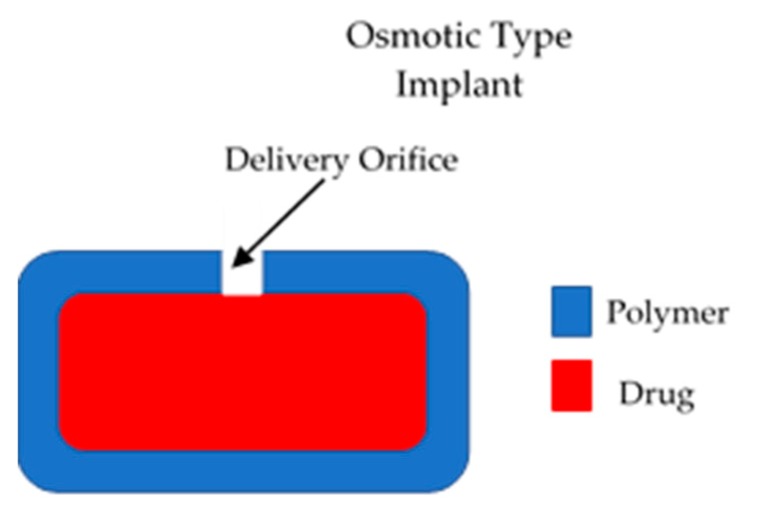
An illustration of an osmotic implantable drug delivery systems.

These types of implants have a positive driving force to control the release of drugs from the device [15]. Therefore, they present a higher degree of control of drug release. However, due to their complexity they present higher development costs [15]. The majority of the implants in this category are electronic systems made of metallic materials. However, to remain within the scope of this article, only polymeric implants will be described. Dynamic drug delivery implants are mainly pump type implants. The main type of polymeric active implants are osmotic pumps. This type of device is formed mainly by a semipermeable membrane that surrounds a drug reservoir [15], as shown in Figure 2. The membrane should have an orifice that will allow drug release. Osmotic gradients will allow a steady inflow of fluid within the implant. This process will lead to an increase in the pressure within the implant that will force drug release trough the orifice. This design allows constant drug release (zero order kinetics). This type of device allows a favourable release rate but the drug loading is limited [15].

## 3. Mechanism of Drug Release from Implantable Polymeric Drug Delivery Systems

Mechanisms of drug release from implantable systems are mainly classified into four groups: matrix degradation; controlled swelling; osmotic pumping; and passive diffusion [19]. For systems based on controlled swelling, solvent penetration into the matrix of the device controls the rate of release. This is usually much slower than diffusion of the drugs, and will, therefore, lead to a lower release rate [15]. Although the diffusion from swollen matrices is mainly responsible for the drug release, matrix degradation could also contribute in the effectiveness of these systems [20].

On the other hand, osmotic pumping and passive diffusion mechanisms of drug delivery are the most promising for linear delivery of drugs. In this case, the amount of released drug is proportional to the square root of the release time.

Osmosis is the overall movement of water from a dilute solution to a more concentrated solution through a partially permeable membrane, and it causes a hydrostatic pressure difference between the two compartments [21]. Osmotic pumping is a phenomenon that utilizes the abovementioned concept to adjust the delivery rate of drugs in defined conditions. In this case, osmotic pressure, caused by water absorption, drives the transport of the drug. Moreover, implantable drug delivery devices based on this phenomenon will demonstrate a constant release rate [22].

Diffusion is a process by which molecules transfer spontaneously from one region to another to equilibrate chemical potential or thermodynamic activity. In this mechanism, migrating molecules are usually known as the diffusants or permeants, and the membrane or matrix in which the diffusant migrates is called the diffusional barrier. Additionally, the external phase is termed medium. The driving force of this drug release mechanism is the concentration gradient or profile of the diffusant within the diffusional barrier [23]. In drug delivery systems mediated by swelling, osmotic pressure or passive diffusion, the release kinetics of drugs will depend on key factors such as: the solubility and diffusion coefficient of the molecule in the polymer; the drug load; and the *in vivo* degradation rate of the polymer [24].

### 3.1. Mechanism of Drug Release from Non-Biodegradable Implants

Non-degradable polymers have been widely applied in the fabrication of transdermal films or implant devices, among other biomedical applications [25]. Representative polymers for the development of these devices include poly(urethanes), silicone, and poly(ethylene vinyl acetate), which will be discussed later in this article. Although multiple types of non-degradable implants are commercially available, membrane enclosed reservoir and matrix-controlled systems (as described previously) are by far the most commonly used [19]. For these non-degradable implants passive diffusion is the main driver of solute transport.

Reservoir-type systems separate a drug compartment from a polymer membrane that presents a diffusional barrier to yield drug flux [23]. These systems have the benefit of maintaining a constant release rate that is not affected by concentration gradient, but most likely is related to the thickness and permeability of the rate-controlling polymeric membrane (zero-order release) [26]. In contrast, matrix-type systems consist of a rate-controlling medium such as a polymer with drug uniformly dissolved or dispersed in it, and typically, a half order drug release corresponds to desorption from the preloaded matrix [23]. In the latter systems, the drug release is mainly mediated by Fickian diffusion, which is affected by concentration gradient, diffusion distance, and the degree of swelling [27,28]. These systems provide a slow diffusion of the drug through the polymeric material, helping to sustain the drug release. Nevertheless, the release kinetics of matrix-type systems are dependent on the volume fraction of the agent in the matrix, meaning that the release from these systems are directly proportional to the encapsulated drug within the matrix [15].

### 3.2. Mechanism of Drug Release from Biodegradable Implants

Implantable drug delivery devices generally consist of a drug reservoir surrounded by a polymer or a drug polymer mixture [6]. When inserted into the desired area of the body, the drug will be released at a pre-determined rate as the polymer degrades. Drug release from a reservoir system is controlled by the rate of polymer degradation or drug dissolution into, and then diffusion through, the polymer wall, or a combination of both. Drug release from a drug polymer mixture is controlled by diffusion, swelling or erosion. The release of drug from the system will be dependent on: the solubility and permeability of the drug in the polymer; the drug load; and the *in vivo* degradation rate of the polymer [6].

Degradation of the polymer and subsequent drug release may occur through one, or a combination of processes including: hydrolysis, in which bonds, e.g., ester bonds, in the polymer backbone are broken down [29]; enzyme degradation, in which hydrolytically susceptible bonds, e.g., amide bonds, demonstrate degradation in the presence of a catalyst [29]; oxidation [30]; and physical degradation, in which bonds are broken as a result of physical forces such as swelling or mechanical loading [30]. The degradation time of polymers can vary extensively depending on features such as polymer molecular weight and surface properties [29]. This will, in turn, affect the release of any drug contained within a formulation. In addition, degradation will also be dependent on *in vivo* factors such as: pH; and temperature [6]. Therefore, the *in vivo* degradation time of any polymer needs to be fully characterised.

## 4. Polymers Used for Implantable Polymeric Drug Delivery Devices

Polymers used to manufacture implantable drug delivery devices can be divided into two categories: biodegradable and non-biodegradable [6]. Major disadvantages of non-biodegradable implants include the need for surgical removal, or accumulation of polymer in the body after use [6]. The surgical removal of non-biodegradable implants is often more traumatic than their insertion [31], as established in a study by Odom et al. [32]. The study investigated the removal of the non-biodegradable contraceptive implant, Nexplanon^®^, and concluded that a multidisciplinary care team and the expertise of a peripheral nerve surgeon may be beneficial to the successful removal of such implants [32]. Alternatively, biodegradable polymers offer the significant advantage of not having to be surgically removed after use. They are designed to degrade naturally to products that can be excreted easily by the body after they have achieved their purpose [29]. The need for surgical removal of an implant made from non-biodegradable polymers complicates its use.

Polymers have been widely investigated for use in tissue engineering and drug delivery. Both natural and synthetic polymers have been investigated [33]. Synthetic polymers are generally biologically inert, have predictable chemical and physical properties, and don’t have the same batch to batch inconsistency that occurs with natural polymers [33,34]. The biodegradability and biocompatibility of any material will be critical when designing a drug delivery system [35]. Any materials used must be fully biocompatible, and any changes in polymer properties that develop as it degrades must be fully investigated and characterised [29].

Ideally, any chosen biodegradable polymer should be: highly reproducible; easily metabolised and excreted by physiological pathways; degradable to non-toxic products; and free from an inflammatory response in vivo [29,36]. A single ideal polymer does not exist, and the choice of polymer will be dependent on the mechanism and rate of release desired, and a combination of polymers may be required to produce the characteristics required.

### 4.1. Biodegradable Polymers

#### 4.1.1. Thermoplastic Aliphatic Polyesters

**Figure 3:**
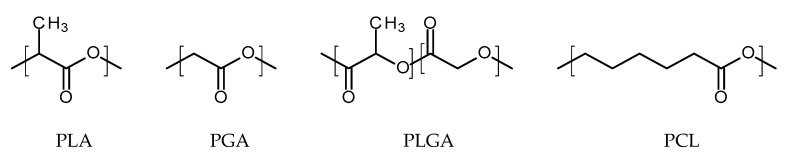
An illustration of the chemical structures of: poly(lactic acid) (PLA); poly(glycolic acid) (PGA); poly(lactic-co-glycolic acid) (PLGA); and poly(caprolactone) (PCL).

**Figure 4:**
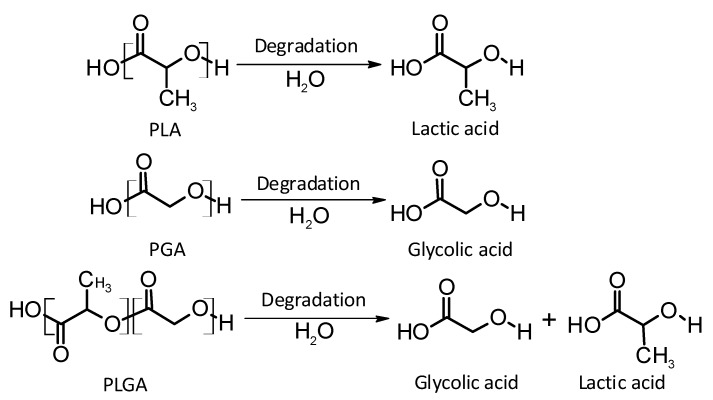
An illustration of the mechanisms of degradation of poly(lactic acid) (PLA); poly(glycolic acid) (PGA); and poly(lactic-co-glycolic acid) (PLGA).

Thermoplastic aliphatic poly(esters) including: poly(lactic acid) (PLA), poly(glycolic acid) (PGA), and poly(lactic-co-glycolic acid) (PLGA) (Figure 3) have been widely investigated due to their favourable characteristics such as biodegradability, biocompatibility and mechanical strength [37,38,39]. These polymers have previously been successfully used in nanoparticle based drug delivery systems and solid and microparticle parenteral implants [38]. Degradation periods for these polymers range from one month to over six months [40]. The mechanisms of degradation for PLA, PGA and PLGA are shown in Figure 4. Degradation rate is affected by factors such as hydrophilicity, glass transition temperature and molecular weight and environmental conditions such as temperature and pH [34,39,40].

##### Poly(lactic acid)

Poly(lactic acid) (PLA) is a biodegradable and bioresorbable polymer that can be obtained through the polymerisation of lactic acid obtained from natural feedstock (i.e., Corn starch rice or potatoes, among others) with promising properties for medical applications [41,42,43,44]. PLA shows similar mechanical properties to other synthetic polymers, such as polypropylene, while presenting lower cost, higher abundance and biodegradability [42]. Moreover, PLA is semipermeable to oxygen and water making this polymer more inclined to biodegradation than other biomedical polymers [43,45,46]. The US Food and Drug Administration (FDA) approved the use of PLA in direct contact with biological fluids as it is a generally recognised as safe (GRAS) material [41]. PLA can be processed using a wide variety of techniques due to its great thermal processability [44]. Accordingly, it can be used in extrusion, film casting, blow moulding or fibre spinning processes, among others [44]. This is a great advantage over other biomaterials such as poly(ethylene glycol). Finally, PLA production requires between 25% and 55% less fossil energy than petroleum-based polymers [44]. Accordingly, PLA is the second most traded polymer in the world [43].

At room temperature PLA is a white powder showing melting and glass transition temperatures of around 175 and 55 °C, respectively [44]. High molecular weight PLA shows similar properties to polystyrene. Due to the existence of two stereoisomers of lactic acid (d and l), PLA can be made using both types of monomers [44]. PLA prepared using d-lactic acid, PDLA, is a crystalline material due to its regular chain structure [44]. On the other hand, PLA made using l-lactic acid, PLLA, will have a hemi-crystalline structure [44]. Additionally, PLA with a mixture of both can be prepared (PDLLA) to obtain an amorphous polymer [44]. All these polymers are soluble in a wide variety of organic solvents such as: benzene, chloroform, acetonitrile, tetrahydrofuran or dioxane [44]. Due to the hydrophobic nature of PLA, this polymer is insoluble in ethanol, methanol and aliphatic hydrocarbons [44].

The biodegradability and mechanical properties of PLA are influenced by the chirality of the monomer. It has been established that d and d/l forms of PLA degrade more rapidly than the l form, as the latter has a higher crystallinity [42,47,48,49,50]. Increasing the surface area-to-volume ratio or the porosity of the polymer will improve the rate of degradation of the polymer [51].

The main PLA mechanism of degradation is the hydrolysis of the ester bond backbone [52]. Therefore, the products obtained in the degradation are lactic acid or lactic acid oligomers. Interestingly, the degradation is catalysed by the newly-formed terminal carboxylic acid groups at the ends of the PLA chains [53]. Temperature and pH influence the degradability of the material. PLA showed higher degradation rates at physiological temperature than at 25 °C. Furthermore, at lower pH the degradation of this polymer is much slower than at physiological pH [54].

In addition to PLA hydrolysis, this polymer can be enzymatically biodegraded. After implantation of the polymer in the body immune cells will be directed to the implantation site. These cells will secrete enzymes, including lactate dehydrogenase and acid phosphatase that will contribute to PLA degradation [55].

##### Poly(glycolic acid)

Poly(glycolic acid) (PGA) is a polyester made by polymerisation of glycolic acid units. It was one of the first biodegradable polymers used for biomedical applications. PGA is a polymer that exists in only one highly crystalline form [47,56]. It exhibits excellent mechanical properties (greater than those of PLA), and a melting point greater than 200 °C [33]. Biodegradable sutures made from PGA have been successfully used, for example, Dexon^®^ [33]. PGA exhibits a quick degradation profile and it is insoluble in many common solvents. Accordingly, this polymer has not been used alone for drug delivery purposes. PGA undergoes bulk degradation *via* scission of its ester backbone to form glycine, which is excreted in the urine or *via* the citric acid cycle [33,47]. However, the acidic by-products of PGA can cause inflammation in the surrounding tissues [47] and limit the potential use of PGA as a lone polymer.

##### Poly(lactic-co-glycolic acid)

Poly(lactic-co-glycolic acid) (PLGA) is a biodegradable and biocompatible copolymer of PLA and PGA [47]. PLGA degrades in the body *via* hydrolysis to form lactic acid and glycolic acid [5,38]. PLGA, therefore, presents itself as an interesting candidate as a polymer for implantable drug delivery devices [40]. It is possible to modify the physical properties of the polymer by altering the polymer molecular weight and ratio of lactide to glycolide [40]. The presence of side methyl groups within PLA make the copolymer more hydrophobic. Thus, PLGA copolymers with high PLA content show higher hydrophobicity and, consequently, a slower degradation rate. Advantages of PLGA include: an increased degradation rate in comparison to PLLA, but decreased in comparison to PDLA; and a lack of acidic by-products produced upon degradation [47]. The monomer composition and the molecular weight of the PLGA molecules have a direct influence in the crystallinity of the polymer. Similar to the previously described polymers, the mechanical properties and the degradation rates are strongly influenced by the degree of crystallinity of the polymer. A higher PGA content within PLGA leads to a lower crystallinity degree and a higher rate of hydration/hydrolysis. PLGA containing 50:50 of PLA:PGA shows the highest degradation rates. PLGA copolymers present *T*_g_ values above 37 °C, thus, exhibiting a fairly rigid chain structure, ideal for implant manufacturing. Finally, PLGA can be processed into a wide variety of shapes and sizes due to its solubility in common solvents such as tetrahydrofuran, acetone, ethyl acetate or chlorinated solvents.

##### Poly(caprolactone)

Poly(caprolactone) (PCL) is a promising candidate for use in polymeric implants due to its biocompatibility, biodegradability, non-toxicity, and relatively low cost [31,33]. It has FDA approval for use in medical applications [57], and it has already been successfully incorporated into materials used for sutures and wound dressings [58]. The presence of unstable aliphatic ester bonds allows the polymer to biodegrade by a mixture of random hydrolysis of ester bonds and bulk degradation pathways [33,38,59,60]. PCL degrades to form products that are metabolised *via* the tricarboxylic acid cycle or are renally eliminated [61]. PCL is a hydrophobic, semi-crystalline polymer [59]. Its low melting point (55–60 °C [33]), good solubility, and good compatibility with other materials make it a promising candidate for use in sub-dermal implants [59].

PCL has a relatively long degradation time, ranging from several months to years, but this is dependent on internal factors, such as its molecular weight [59], and degradation conditions such as: temperature; pH; and presence of enzymes. Degradation time increases as molecular weight increases. As molecular weight increases, the chain length and, therefore, the number of ester bonds that need to be cleaved to create water soluble monomers and oligomers also increases [59]. The slow degradation of PCL is due to its hydrophobic nature, which does not allow water penetration [1,60]. The rate of hydrolysis of ester linkages, therefore, will be affected by factors which increase the penetration of water into the polymer [57].

PCL degrades slower than other polymers such as PGA, PLA and PLGA [61]. However, PCL is a relatively low-cost polymer, which may make it more commercially viable for use in a product. As a result of this comparatively long degradation time, co-polymers of PCL have been investigated [33]. PCL has been shown to be compatible and forms miscible blends [33] with other polymers. This provides the opportunity to create polymer blends with unique properties and degradation kinetics. For example, copolymerisation with hydrophilic monomers can increase the rate of degradation [61,62]. Poly(ethylene glycol) (PEG), with its inherent hydrophilicity and lack of immunogenicity and toxicity, is a promising candidate for copolymerisation with PCL to form a material with improved hydrophilicity and biodegradability [4]. PCL-PEG copolymers have been shown to have increased biocompatibility when compared to the PCL homopolymer [4]. The addition of PEG to PCL is likely to reduce the degradation time of the polymer by allowing increased water penetration and, therefore, rate of hydrolysis. The polymer’s glass transition temperature (*T*_g_) and crystallinity will also affect the ability of water to penetrate the polymer [57]. A high *T*_g_ will correlate to limited molecular motion, low free volume within the polymer and, therefore, reduced availability for water penetration. Reduction of *T*_g_ and crystallinity will accelerate hydrolytic degradation. Addition of PEG to form PCL-PEG blends is likely to create a blend with lower *T*_g_ and crystallinity and, therefore, reduced degradation time. The rate of degradation can also be altered by copolymerisation with other lactones, glycolides or lactides [59]. PCL, therefore, is a highly diverse material and has the potential to be a suitable polymer in the development of implantable drug delivery systems.

##### Other Biodegradable Polymers

PLA, PGA, PLGA and PCL are the most commonly used biodegradable polymers for biomedical applications because of their biocompatibility, mechanical strength and ease of formulation [63]. However, numerous less commonly used biodegradable polymers for drug delivery exist including: poly(amides), poly(anhydrides), poly(phosphazenes) and poly(dioxanone) [34,64]. Poly(anhydrides) have a low hydrolytic stability resulting in rapid degradation rates, making them suitable for use in short-term controlled delivery systems [65]. Poly(phosphazenes) have a degradation rate that can be finely tuned by appropriate substitution with specific chemical groups and use of these polymers has been investigated for skeletal tissue regeneration and drug delivery [65]. Poly(dioxanone), like PCL, is a polylactone that has been used for purposes such as drug delivery, and tissue engineering [66].

### 4.2. Non-Biodegradable Polymers

#### 4.2.1. Poly(siloxanes)

**Figure 5:**
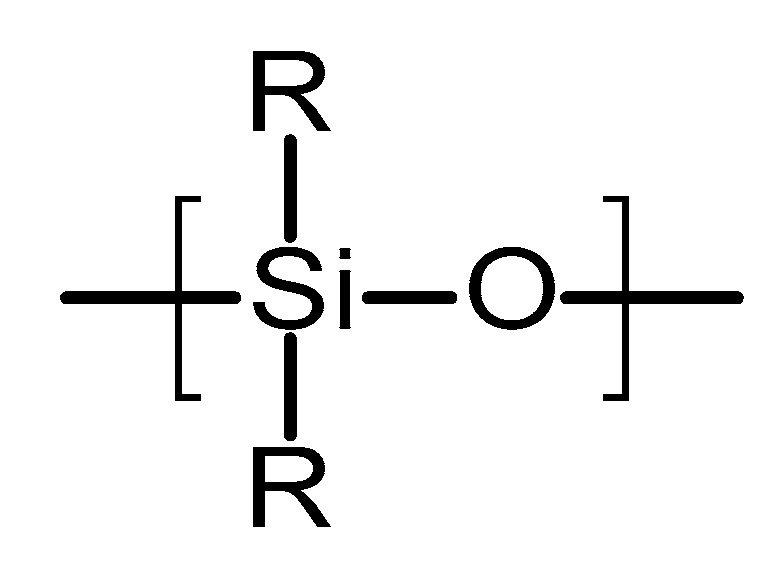
An illustration of the chemical structure of poly(siloxane).

Poly(siloxanes) or silicones are organosilicon polymeric materials composed of silicon and oxygen atoms [67]. Figure 5 shows the chemical structure of this type of polymer. Lateral groups can be methyl, vinyl or phenyl groups [67]. These groups will influence the properties of the polymer. Poly(siloxanes) have been extensively used in medicine due to the unique combination of thermal stability, biocompatibility, chemical inertness and elastomeric properties [67]. The silicones commonly used for medical devices are vulcanised at room temperature. They are prepared using a two-component poly(dimethylsiloxanes) (PDMS) in the presence of a catalyst (platinum based compound) [67,68]. The final material is formed *via* an addition hydrosilation reaction [67]. An alternative method to obtain silicones for medical applications is the using linear PDMS with hydroxyl terminal groups [69]. This linear polymer is cross-linked with low molecular weight tetra(alkyloxysilane) using stannous octoate catalyst.

Non-biodegradable polymers show different drug release mechanisms than biodegradable polymers [26]. The latter relies on two main factors: the diffusion of the drug from the matrix and the degradation of the matrix. This topic is discussed in a previous section of this work. However, non-biodegradable polymers such as PDMS rely solely on the diffusion of the drug from a PDMS matrix or through a PDMS membrane [26,67]. PDMS has been used as a rate controlling membrane in a wide variety of drug delivery devices [26,67]. Therefore, the diffusivity of the drug through the PDMS is crucial to control the dose [26]. PDMS is prepared as a crosslinked network. As a result of this, the crosslinking density will influence water uptake of the material and the diffusivity of the drug [26]. Accordingly, these parameters should be optimised in order to obtain the required drug release profile.

#### 4.2.2. Poly(ethylene-vinyl acetate)

**Figure 6:**
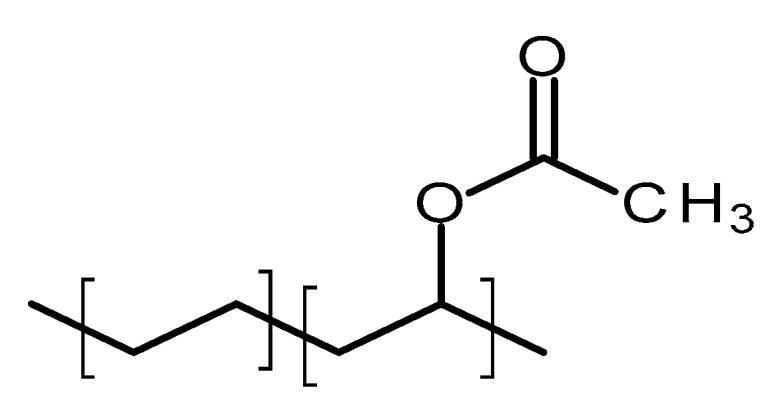
An illustration of the chemical structure of poly(ethylene-vinyl acetate) (PEVA).

Poly(ethylene-vinyl acetate) (PEVA) is a thermoplastic copolymer of ethylene and vinyl acetate [70]. The units of these two monomers are randomly distributed through the polymer chain [71]. Figure 6 shows the structure of this polymer. The ratio between the two monomers will strongly influence the properties of the polymer [71]. The vinyl acetate (VA) content will impact on the melting point, crystallinity and stiffness of the material [71]. The crystallinity of PEVA decreases when the content of VA increases [71]. Accordingly, the final properties of the material can be tailored by optimising the monomers ratio. The biocompatibility of PEVA has been extensively demonstrated and it was approved by the FDA [70]. Additionally, it is on the inactive ingredients list for non-drug products [70]. Finally, PEVA is a non-degradable/resorbable material [13].

PEVA has been extensively used for drug delivery applications [13,70]. As PEVA is a non-degradable material, the diffusion of the drug from the matrix will govern the release mechanism [26]. However, PEVA can be used in reservoir type systems as rate controlling membrane [13,70]. Increasing the crystallinity reduce the diffusivity of the polymer. Accordingly, by changing the vinyl acetate/ethylene ratio can tailor the drug release profile [26].

### 4.3. Other Polymers

#### 4.3.1. Poly(urethanes)

**Figure 7:**
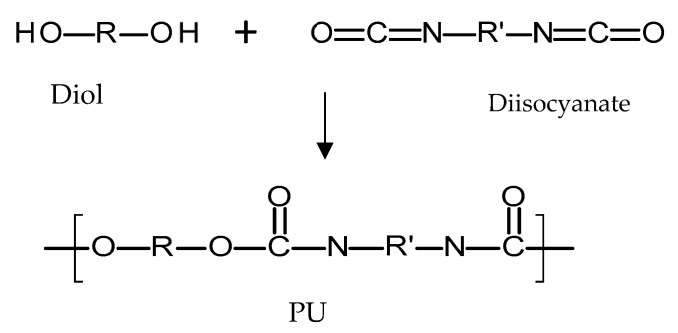
An illustration of the chemical structure of poly(urethane) (PU).

Polyurethanes (PU) are a broad family of polymers obtained from the reaction of diisocyanates with polyols using a catalyst, as seen in Figure 7 [72]. This type of polymer presents a wide variety of structures and properties, as different polyols/diiscyanates can be used to synthesize this type of polymer [72]. The diisocyanates contain two –N=C=O groups per molecule [73] and they can be aliphatic, mono/polycyclic or aromatic [72]. The use of different isocyanates will influence the properties of the resulting polymer [72]. Aromatic isocyanates will lead to more rigid materials with lower oxidative stabilities [73]. Aliphatic isocyanates will present better oxidative stability [73]. On the other hand, different polyols can be used to obtain PU such as: polyesters, polyester polyols, polycaprolactones, polycarbonates and polyethers [72]. Finally, there is an alternative type of compound added during PU synthesis that changes the final properties of the material: chain extenders [72,74]. These molecules are normally short-chain diols such as 1,4-butanediol. PU polymers are normally formed by hard segments and soft segments [72,75]. The soft segments are dependent on the diol molecule chain and provide flexibility to the material [72,75]. On the other hand, the hard segments are formed by the reaction between the chain extenders and the diisocyanates and they provide extra strength to the material [72,75]. Due to the variety of the parameters that can be modified, PUs are a broad family of polymers [76]. Biodegradable PU can be prepared by using biodegradable polyols such as poly(caprolactone) or poly(ethylene glycol) [77]. On the other hand, PU can be prepared to obtain PU based rubbers with similar properties and behaviour to PDMS [67]. Accordingly, this type of polymer is versatile.

#### 4.3.2. Natural Polymers

In addition to the biopolymers, such as the abovementioned PLA, there a few natural polymers which also represent a promising class of materials with a wide range of applications, including use in implantable devices. These natural polymers include, cellulose, chitosan, silk and others naturally derived proteins. These materials present certain advantages compared to the traditional materials (metals and ceramics) or synthetic polymers, such as biocompatibility, biodegradation and non-cytotoxicity, which make them ideal to be used in implantable drug delivery devices [78].

##### Cellulose

**Figure 8:**
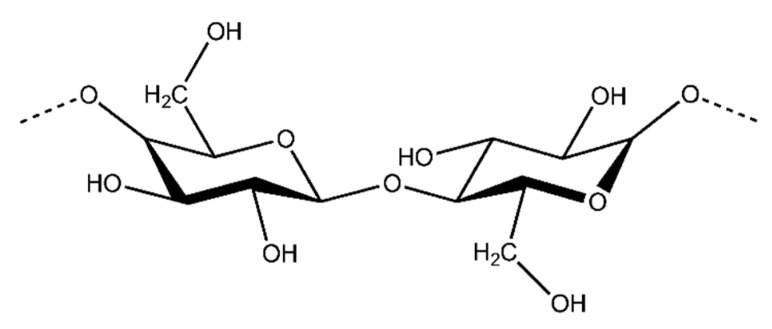
An illustration of the chemical structure of cellulose [80].

Cellulose is the most abundant organic compound in the world and it is mostly produced by plants. Cellulose is a natural linear polymer (polysaccharide), whose structure consists of long polymer chains of repeating β-d-glucopyranose units that are covalently linked through acetal functions between the C_1_ carbon atom and the equatorial −OH group of C_4_ (β-1,4-glycosidic bonds) (Figure 8) [79].

Cellulose and its many derivatives, including cellulose ethers/esters, micro/nano-sized cellulose products, bacterial cellulose (BC), have been widely studied for many applications [81,82,83,84,85]. For instance, Modulevsky et al. used the native hypanthium tissue of apples to create implantable cellulose scaffolds [84]. This approach is complementary to the use of BC which has been successfully employed for the development of implantable materials. Due to its nanostructure and properties, BC is a promising candidate for a great range of medical applications. BC fibres have a high degree of crystallinity and their endotoxin level is within the suitable range of endotoxin values for implants based on the FDA, which means they could be used safely in intravenous applications. BC has been shown to possess more suitable properties than plant-derived cellulose or nanocellulose and it is mainly due to its biosynthesis procedure [81,83]. It is important, however, to note that plant-derived polymers are much more cost effective to produce and are extremely straightforward to prepare for implantation.

Additionally, cellulose nanocrystals (CNCs) and cellulose nanofibrils (CNFs) are also currently under intense investigation for the development of biomedical applications such as: implants, tissue engineering, drug delivery, antibacterial/antimicrobial, cardiovascular and wound healing [85]. Indeed, it can be observed how the number of publications in the period 2000–2013 on cellulose materials for such applications has been gradually increasing [85].

##### Chitosan

**Figure 9:**
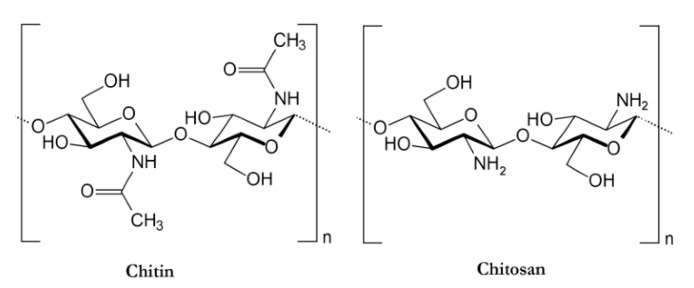
An illustration of the chemical structures of Chitin and Chitosan [90].

Chitosan is the second most abundant polymer, after cellulose. It is a cationic polysaccharide produced by deacetylation of chitin, which is found in the exoskeletons of insects, the cell walls of fungi, and certain hard structures in invertebrates and fish [78,86]. This natural polymer is structurally composed of *N*-acetyl-d-glucosamine and d-glucosamine units with one amino (NH_2_) group and two hydroxyl (–OH) groups in each repeating glycosidic units [87]. The structure of chitosan is shown in Figure 9. Due to its properties, it has been extensively used in a large amount of applications ranging from medical to industrial areas, including application in implantable drug delivery devices [87,88,89].

##### Silk

Silk is another commonly natural polymer used to develop implantable drug delivery devices. It is assumed that the largest producer of silk in nature is the silkworm, *Bombyx mori* [91]. Raw silk coming from a silkworm is composed of fibroid in the core; silk fibroin, which is the structural protein produced in the posterior region of the *Bombyx mori* gland; and a glue-like coating consisting of sericin proteins [78].

**Figure 10:**
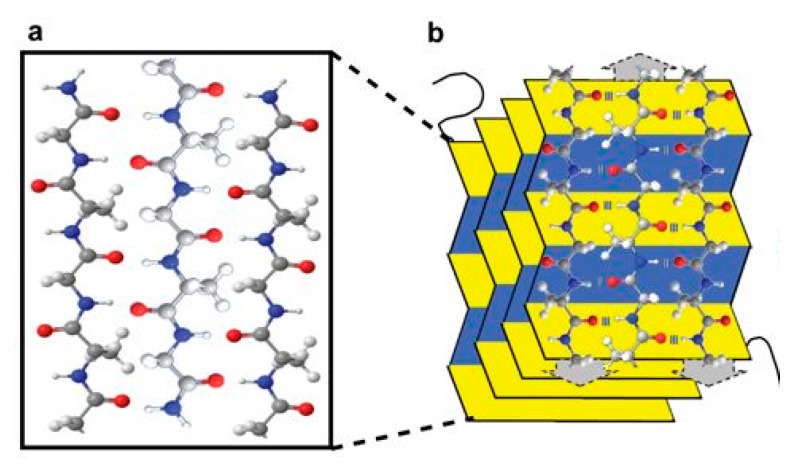
An illustration of (**a**) a three chain silk sequence composed of carbon (gray), nitrogen (blue), oxygen (red) and hydrogen (white); (**b**) 3-dimensional β-sheet of silk. Reproduced with permission from [100].

The structure of silk fibroin has been well-characterised, and some recent studies suggest that this structural protein shows exceptional physicochemical and biological properties desirable for drug delivery applications [91]. Structurally, silk fibroin forms a β-sheet structure in which hydrogen bonds and van der Waals interactions generate a structure that is thermodynamically stable (Figure 10) [78]. Therefore, silk is a versatile natural polymer that can be used for various drug delivery applications including: injectable particles [92,93], bioadhesives [94,95], hydrogels [96,97], reservoirs and scaffold implants [98,99].

Nowadays, other naturally derived proteins such as: collagen, gelatin, albumin, elastin and milk proteins have led the research community to offer an interesting alternative to PLGA-based systems and are being extensively investigated for their potential use in drug delivery applications [101].

## 5. Methods of Implant Manufacture

A number of factors need to be considered when choosing a manufacturing method for production of an implantable drug delivery devices including: cost, efficiency and differences in properties of the produced implants. Implants can be manufactured using a variety of techniques including: compression, solvent casting, hot melt extrusion, injection moulding or more recently 3D printing. Thermoplastic polymers such as PLA or PLGA can produce implants using techniques such as: hot moulding, injection moulding, compression or extrusion [7]. Implants prepared by different techniques are unlikely to form polymers with exactly the same microporous structure and will degrade at different rates and, therefore, will have different *in vitro* and *in vivo* release profiles [7]. Fialho et al. compared the process of hot moulding and compression as techniques to make intra-ocular implants, and found that, the manufacturing technique significantly influenced the polymer degradation and, therefore, drug release from the resulting implants [7], with compressed implants showing an increased rate of drug release than their moulded counterparts.

### 5.1. Compression

One advantage of compression as a manufacturing technique is the lack of requirement for use of heat or solvents, making it a suitable method for manufacture of implants containing heat or solvent sensitive compounds such a proteins or peptides [102]. However, implants produced using this technique often show a faster release profile than observed with other manufacturing techniques, and drug release may need to be prolonged using additional methods, such as coating the implant. In addition, as shown by Fialho et al., implants produced by compression had an irregular surface with many pores and channels [7], which may lead to irregular release from implant produced in this way.

### 5.2. Solvent Casting

In the solvent casting method, the polymer is first dissolved in a suitable solvent, then the resulting solution is cast into a mould and the solvent is removed by evaporation [40]. Implants produced by this method often result in films or laminar implants [103,104,105]. A disadvantage of this method is the need for large amounts of organic solvent, which can have an effect on the stability of drugs and toxicity, and may give rise to environmental concerns [40].

### 5.3. Hot Melt Extrusion

Hot melt extrusion is the process of melting, mixing, and forcing a polymer through a small orifice called a die [40]. A prerequisite for the use of melt extrusion is that the polymers used must be thermoplastic [106]. Aliphatic poly(esters) including PLA, PGA and PLGA are all thermoplastic and, therefore, suitable for processing by this method [106]. This method offers the advantage of requiring no solvents; however, it can cause the degradation of thermally labile drugs [40]. This does not preclude its use in manufacture of implants containing thermally labile drugs. Repka et al. found that it was possible to successfully incorporate hydrocortisone, a thermally labile drug, into hydroxypropyl cellulose (HPC) films produced by melt extrusion [107]. Products such as Zoladex^®^, Depot-Profact^®^ and Implanon^®^ are manufactured in this way using melt extrusion [7,106]. Extrusion can be performed as a continuous process, which allows high throughput rates [106].

### 5.4. Injection Moulding

Thermoplastic polymers such as PLGA or PLA can be manufactured into implants using injection moulding. The polymer is heated, injected into a specific mould and allowed to solidify. As a result of the high heat applied, a decrease in the molecular weight of the polymers can be seen. The effect of manufacturing using extrusion versus injection moulding on the degradation properties of a polymeric matrix of PLA was investigated by Rothen-Weinhold et al. [108]. It was found that the molecular weight and polydispersity was reduced by both techniques, but the decrease was more pronounced with injection moulding. As a result, extruded implants degraded more rapidly than those manufactured using injection moulding [108].

### 5.5. 3D Printing

3D printing technology is currently used to produce dental implants, protheses and orthopaedic implants [109]. It is a cost-effective, reproducible and highly adaptable method and could be very promising in the manufacture of implantable drug delivery devices [109]. 3D printing could be used to manufacture the biodegradable implant structure, which would subsequently be filled with the drug, with release from the implant controlled by degradation of the implant structure, or rate-controlling membranes covering orifices in the implant. 3D printing is an extremely promising technique and would be particularly valuable in the rapid production of prototypes for investigation. Its suitability for use as a mass production manufacturing technique is still uncertain. However, the suitability of 3D printing for the manufacture of commercial products took a step forward in 2015 after the FDA approval of a 3D-printed drug product [110].

## 6. Implantable Polymeric Device Design

**Figure 11:**
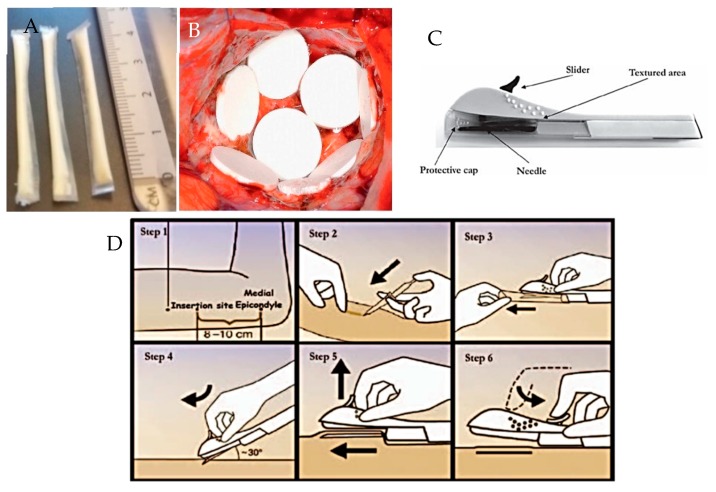
Images showing: (**A**) prototype PCL film implants [9]; (**B**) Gliadel Wafers^®^, reproduced with permission from [114] (**C**) Nexplanon^®^ applicator device and (**D**) insertion of Nexplanon^®^ using its applicator device, reproduced with permission from [115].

Implantable polymeric rods are the most widely used design [5,111]. Other implantable drug delivery device designs exist, for example, those which are formed *in situ* as a result of a polymer undergoing a sol-gel transition, for example Oncogel^®^ [112]. Newer polymeric designs under investigation include: PCL film implant devices, as shown in Figure 11A [9]; and silicone rods with dissolving membranes [113]. Advances in manufacturing techniques may result in the creation of more complex implant designs to allow very specific drug release targeted to a specific disease or individual patient requirements.

As the majority of implantable drug delivery devices are rod shaped and are designed to be delivery sub-cutaneously or intramuscularly, the most common method of implantation is *via* a needle or surgical implantation. Some implants, for example Nexplanon^®^, are inserted *via* a specifically designed applicator device that comes with the product, as shown in Figure 11C,D. Other sites for implantation include: intravaginal, intraocular, intra-vesicular, and intra-tumoral.

## 7. Current Therapeutic Applications

Implantable drug delivery devices have the potential to be used for a wide variety of clinical applications in areas including, but not limited to: women’s health, oncology, ocular disease, pain management, infectious disease and central nervous system disorders [6,15]. Examples of implantable drug delivery devices for each of these areas are summarised in Table 1, Table 2, Table 3 and Table 4.

Women’s health is one area where implantable drug delivery devices have had a large impact, particularly in their use for contraception. In 1990, Norplant became the first implantable contraceptive device to be approved. Implantable long acting contraceptives have been shown to be the among the most effective form of contraception, with an annual pregnancy rate of less than 1% for women using these methods [116,117]. Table 1 shows examples of implantable drug delivery devices for use in women’s health.

Systemic delivery of chemotherapeutic agents is the most common route of administration. However, it often involves delivery of the agents at their maximum tolerated dose which can lead to severe side-effects such as neutropenia and cardiomyopathy [125]. Implantation of a drug delivery device close to the site of action may allow reduced systemic exposure and as a result reduce the damage caused to healthy tissue. Some examples of implantable drug delivery devices for the treatment of cancer are shown in Table 2.

Drug delivery to the posterior segment of the eye is difficult due the unique anatomical and physiological barriers that the ocular environment presents [133]. Successful treatment of ocular conditions requires that the dose of drug or therapeutic agent is delivered to the site of action and retained for the duration that the treatment is required. This is particularly challenging in the eye because of poor drug permeation and poor drug retention in the eye due to lacrimation, tear dilution and tear turnover [134]. These issues are also compounded by poor patient compliance and difficult device use associated with ocular conditions [134,135]. Implantable drug delivery devices overcome some of these challenges to delivery by reducing the number of treatment applications required, but also come with their own challenges including: burst release, the possibility of dose dumping, and low bioavailability [134]. Table 3 highlights some examples of implantable ocular devices that have been developed.

The use of implantable drug delivery devices for pain management is promising. Chronic pain is particularly difficult to treat and is associated with a high risk of addiction or death from overdosing. Implantable drug delivery devices have the potential to be of use in infectious diseases, and in particular tuberculosis (TB). The treatment for TB is long and the drugs used are associated with side-effects. These factors result in poor patient compliance with the treatment regimen and often leads to treatment failure and the development of resistance. In this context, an implantable drug delivery device would be ideal to ensure patient compliance and completion of the treatment. Poor patient compliance to antipsychotic therapy is a common occurrence and causes a high risk of relapse, hospitalisation and other negative outcomes [139]. About 50% of patients with schizophrenia are thought to be non-compliant with their prescribed medication [140]. Parenteral administration of antipsychotics offers advantages such as: increased bioavailability, lower drug serum levels and decreased variation in drug plasma levels [140]. As well as these advantages, use of a long-acting implantable drug delivery device would ensure 100% patient compliance. An overview of some examples of implantable drug delivery devices used for pain management, infectious diseases and central nervous system disorders are summarised in Table 4.

## 8. Conclusions

The market for polymeric implantable drug delivery devices is one that is growing. The advantages that this delivery route demonstrate over more conventional drug delivery methods, such as oral tablets, make it likely that it will continue to grow and that the number of implantable drug delivery devices on the market will increase. However, implantable drug delivery devices have a number of disadvantages including the invasive nature of this delivery method. The advantages that these devices can offer with respect to patient compliance, stability of drugs within these devices and removability if adverse reactions occur, outweigh these disadvantages that exist. Current therapeutic applications of implantable drug delivery devices are covered in this article. However, the use of implantable drug delivery devices has the potential to span far greater than these conditions mentioned. One such condition where these devices could have a major impact is in the treatment or prevention of human immunodeficiency disease (HIV). 3D printing offers an interesting prospect as an exciting new manufacturing method, one which provides a unique opportunity to produce complicated designs or personalised implantable devices. However, when compared to more traditional methods of implantable device manufacture, such as hotmelt extrusion or compression moulding, this manufacturing method comes with additional scale up and regulatory challenges. The FDA approval of the first 3D printed tablet in 2015 makes the reality of 3D printing as a pharmaceutical manufacturing method much more likely.

## Figures and Tables

**Table 1:** Examples of implantable drug delivery devices used in the area of women’s health.

Product Name	Implant Type	Material	Drug Delivered	Indication	References
**Norplant^®^**	Sub-cutaneous	Silicone	Levonorgestrel	Contraception	[118,119]
**Jadelle^®^**					
**Estring^®^**	Intra-vaginal	Silicone	Estradiol	Menopausal symptoms	[120]
**Nuvaring^®^**	Intra-vaginal	PEVA	Etonogestrel, Ethinyl estradiol	Contraception	[121,122]
**Implanon^®^**	Sub-cutaneous	PEVA	Etonogestrel	Contraception	[123,124]
**Nexplanon^®^**					

**Table 2:** Examples of implantable drug delivery devices used for anticancer therapy. ND = not disclosed.

Product Name	Implant Type	Material	Drug Delivered	Indication	References
**Zoladex^®^**	Sub-cutaneous	PLGA	Goserelin	Prostate cancer	[126]
**Prostap^®^SR**	Sub-cutaneous	PLGA	Leuprolide	Prostate cancer	[127]
**Gliadel Wafers^®^**	Intra-tumoral	Silicone	Carmustine (BCNU)	Primary malignant glioma	[114,128]
**Oncogel^®^**	Intra-tumoral	PLGA-PEG-PLGA	Paclitaxel	Oesophageal cancer	[129]
**Vantas^®^**	Sub-cutaneous	Methacrylate based hydrogel	Histrelin	Prostate Cancer	[130,131]
**GemRIS^®^**	Intra-vesical	ND	Gemcitabine	Non-muscle invasive Bladder Cancer	[132]

**Table 3:** Examples of implantable drug delivery devices used to treat ocular diseases.

Product Name	Implant Type	Material	Drug Delivered	Indication	Reference
**Ocusert^®^**	Intra-ocular	PEVA	Pilocarpine, Alginic acid	Open angle glaucoma	[136]
**Retisert^®^**	Intra-ocular	Microcrystalline cellulose, PVA, Magnesium stearate	Fluocinolone	Non-infectious uveitis	[137]
**Vitrasert^®^**	Intra-ocular	PVA, PEVA	Ganciclovir	CMV retinitis in AIDS patients	[138]

**Table 4:** Examples of implantable drug delivery devices for pain management, infectious disease and central nervous system disorders. ND = Not disclosed.

Therapeutic Indication	Product Name	Implant Type	Material	Drug Delivered	Indication	References
**Pain**	ND (Axxia Pharmaceuticals)	Sub-cutaneous	PU, PEG/PPG/PTMEG	Hydromorphine	Chronic neuropathic pain	[141]
	LiRIS^®^	Intra-vesical	Silicone	Lidocaine	Interstitial cystitis/bladder pain syndrome	[142,143]
	Probuphine^®^	Sub-cutaneous	PEVA	Buprenorphine	Opioid abuse	[144]
**Infectious Diseases**	ND	ND	PLGA	Isoniazid	TB	[145]
	ND	ND	PLGA	Isoniazid, Pyrazinamide	TB	[146]
**Central Nervous System disorders**	Med-Launch	Sub-cutaneous	PLGA	Risperidone	Schizophrenia	[8,147]
	ND	Sub-cutaneous	PU	Risperidone	Schizophrenia	[148]
	Risperdal consta^®^	Intra-muscular	PLGA	Risperidone	Schizophrenia	[149]
